# The Interface between Cell Signaling Pathways and Pregnane X Receptor

**DOI:** 10.3390/cells10113262

**Published:** 2021-11-22

**Authors:** Robert S. Rogers, Annemarie Parker, Phill D. Vainer, Elijah Elliott, Dakota Sudbeck, Kaushal Parimi, Venkata P. Peddada, Parker G. Howe, Nick D’Ambrosio, Gregory Ruddy, Kaitlin Stackable, Megan Carney, Lauren Martin, Thomas Osterholt, Jeff L. Staudinger

**Affiliations:** 1 Division of Basic Sciences, Farber-McIntire Campus, College of Osteopathic Medicine, Kansas City University, Joplin, MO 64804, USA; rrogers@kansascity.edu (R.S.R.); aparker@kansascity.edu (A.P.); phill.vainer@kansascity.edu (P.D.V.); elijah.elliott@kansascity.edu (E.E.); dakota.sudbeck@kansascity.edu (D.S.); vpeddada@kansascity.edu (V.P.P.); parker.howe@kansascity.edu (P.G.H.); gregory.ruddy@kansascity.edu (G.R.); kstackable@kansascity.edu (K.S.); megan.carney@kansascity.edu (M.C.); lauren.martin@kansascity.edu (L.M.); thomas.osterholt@kansascity.edu (T.O.); 2 Thomas Jefferson Independent Day School, Joplin, MO 64801, USA; kaushal.parimi@gmail.com

**Keywords:** nuclear receptor, pregnane X receptor, xenobiotics, cell signaling, phosphorylation, SUMOylation, ubiquitination, acetylation, PARylation

## Abstract

Highly expressed in the enterohepatic system, pregnane X receptor (PXR, NR1I2) is a well-characterized nuclear receptor (NR) that regulates the expression of genes in the liver and intestines that encode key drug metabolizing enzymes and drug transporter proteins in mammals. The net effect of PXR activation is to increase metabolism and clear drugs and xenobiotics from the body, producing a protective effect and mediating clinically significant drug interaction in patients on combination therapy. The complete understanding of PXR biology is thus important for the development of safe and effective therapeutic strategies. Furthermore, PXR activation is now known to specifically transrepress the inflammatory- and nutrient-signaling pathways of gene expression, thereby providing a mechanism for linking these signaling pathways together with enzymatic drug biotransformation pathways in the liver and intestines. Recent research efforts highlight numerous post-translational modifications (PTMs) which significantly influence the biological function of PXR. However, this thrust of research is still in its infancy. In the context of gene-environment interactions, we present a review of the recent literature that implicates PXR PTMs in regulating its clinically relevant biology. We also provide a discussion of how these PTMs likely interface with each other to respond to extracellular cues to appropriately modify PXR activity.

## 1. Introduction

Pregnane X receptor (NR1I2, PXR) is a liver- and intestine-enriched member of the nuclear receptor (NR) superfamily of ligand-activated transcription factors. Based upon its conserved DNA sequence homology with other previously identified NRs, several groups cloned PXR in 1998 [1,2,3,4]. Subsequent combinatorial chemistry and high-throughput screening approaches led to the discovery that structurally diverse steroids and a myriad of clinically used pharmaceutical drugs activate this novel nuclear receptor superfamily member. The PXR protein is now known as a master-regulator of drug-inducible gene expression of an assemblage of functionally linked gene products in mammalian livers and intestines. The knowledge that PXR activators all regulate the drug-inducible expression of specific cytochrome P450 (CYP) enzymes and transmembrane drug transporter proteins solved a years-old clinical observation in which administration of certain drugs induce their own metabolism, and that of numerous other co-administered drugs in patients on combination therapy [5,6]. The CYP family of enzymes are monooxygenases involved in the hydroxylation of steroids including corticosteroids, androgens, progestins, dehydroepiandrosterone, as well as foreign compounds including numerous xenobiotics and clinically used drugs [7,8]. The first identified, and now canonical, PXR-target genes in the liver were *CYP3A4* and *CYP2B6* [9,10,11,12,13]. The expression of the *CYP3A4* gene in humans is induced by a variety of compounds and drugs including xenobiotics, glucocorticoids, anti-glucocorticoids (e.g., RU486, PCN), macrolide antibiotics (e.g., rifampicin), imidazole antifungals (e.g., clotrimazole), HMG-CoA reductase inhibitors (e.g., lovastatin), and barbiturates (e.g., phenobarbital). More recently, data have emerged revealing that intestinal epithelial cells are exposed to microbial-derived ligands derived from tryptophan and its gut-specific microbial metabolite indole 3-propionic acid, which activate PXR to function as a central regulator of the gastro-intestinal barrier function [14]. This regulation is intrinsically associated with intestinal bacteria involved with the metabolism of tryptophan and the production of indoles such as indole 3-propionic acid that are known to activate PXR in intestinal epithelial cells.

In general, the enzymes and transmembrane transporters that are up-regulated following PXR activation comprise a network of proteins that collaboratively function in the oxidative metabolism and transport of numerous lipid-soluble xenobiotic and endobiotic compounds, thereby converting these compounds into more water-soluble molecules for eventual elimination from the body [15,16,17,18]. PXR was thus initially defined as a “xeno-sensor” that regulates a key protective response in our enterohepatic system [19]. On the other hand, PXR activation represents the molecular mechanism of a class of clinically important drug–drug interactions in which one compound vastly accelerates the metabolism of other pharmacotherapy in patients on certain combination therapies.

Outside of the liver, PXR has been shown to regulate P-glycoprotein in enterocytes and other endothelial cell types involved in the gut–blood and blood–brain barriers [20,21,22]. PXR activation has also been implicated in the response to chemotherapy in reproductive tissues such as ovaries [23,24,25,26,27]. This tissue distribution is in line with PXR’s role in xenobiotic and drug metabolism. Furthermore, the role of PXR as a nuclear receptor has evolved to include its regulation of families of genes involved in steroid hormone homeostasis, bile acid metabolism, oxidative stress, inflammation, cell proliferation, and glucose and lipid metabolism [8,9,16,28,29,30]. More recent research efforts reveal that PXR influences the expression of hundreds of genes, providing key evidence that PXR likely influences a wide variety of cellular processes [31].

While it was initially believed that PXR exclusively resided in the nucleus where it performed its role as a transcriptional regulator, it has since been shown that ligand-dependent translocation of PXR from the cytoplasm to the nucleus occurs [32,33]. In the absence of ligand, PXR resides in the cytoplasm in a multi-protein complex with CAR-cytoplasmic-retention-protein (CCRP) and heat shock protein 90 (HSP90) [33]. Upon ligand binding, PXR undergoes a conformational change that promotes dissociation of the cytoplasmic retention co-chaperone complex. Thus, PXR translocates to the nucleus where it interfaces with its obligate partner, the retinoid acid X receptor-alpha (RXRα), to direct genetic programs comprising a xenobiotic response of transactivation and transrepression of both direct and indirect PXR-target genes, respectively.

**Figure 1:**
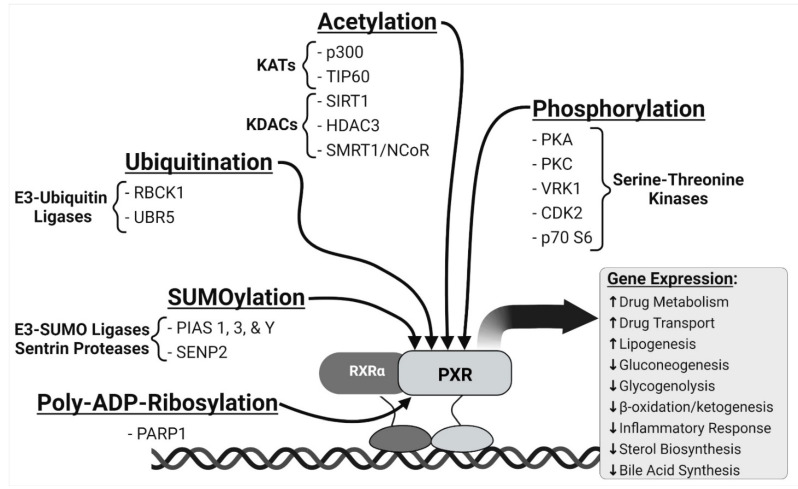
A pictorial diagram of the types of post-translational modification of pregnane X receptor (PXR, NR1I2). Created with BioRender.com (10 November 2021).

The transactivation capacity of NR proteins is mediated by an exquisite and highly choreographed interface with multi-protein enzyme complexes and scaffold proteins to alter post-translational modifications (PTMs) of chromatin-associated regulatory proteins [34]. Specific co-activator proteins that collaborate with PXR to modulate target-gene expression include steroid receptor coactivator 1 (SRC1), SRC2, p300, and peroxisome proliferator activated receptor gamma coactivator 1 alpha (PGC1α) [3,4,35,36,37,38]. The co-repressor proteins known to physically associate with PXR include the nuclear receptor co-repressor (NCoR) and silencing mediator for retinoid and thyroid receptors (SMRT), histone deacetylase 3 (HDAC3), and SIRT1 [35,39,40,41,42]. In addition, several PTMs of PXR have been observed or inferred that modulate PXR biological activity and sub-cellular location to include phosphorylation [43,44,45,46,47,48,49,50,51], ubiquitylation [52,53,54,55], SUMOylation [56,57,58], acetylation [55,57,59,60], and poly(ATP-ribosyl)ation [59]. A series of experimental studies, largely based upon consensus in silico kinase site prediction analysis and direct observation, have been performed in an effort to identify the interface between PXR and pivotal cell signaling pathways (Figure 1). The focus of this review will be on how these PTMs influence each other to coordinate the transactivation capacity of PXR and the subsequent alterations in cellular biochemical processes that occur as a result.

## 2. PXR and Cell Signaling Pathways

### 2.1. Phosphorylation

Much of the PXR-mediated xenobiotic response may be governed initially by kinase signaling pathways that terminate at the level of phosphorylation of the PXR protein. Initial efforts at examining PXR interface with kinase signaling cascades included the use of a protein kinase C (PKC) activator, phorbol 12-myristate 13-acetate, to show that PKC signaling repressed PXR activity in cell-based reporter gene assays and in cultured hepatocytes [44]. A subsequent study by this group unveiled the effects of protein kinase A (PKA) signaling on PXR biology using a diterpene compound called forskolin [60]. Forskolin is a molecule that is classically used to directly activate the PKA signaling pathways in cell-based assays. These authors revealed that activation of the PKA signaling pathway potentiates PXR-mediated induction of *Cyp3a11* gene expression in cultured mouse hepatocytes and increases the strength of PXR-coactivator protein–protein interaction in cell-based reporter gene assays. Moreover, using in vitro kinase assays, they revealed that purified PXR can serve as a substrate for catalytically active PKA using in vitro phosphorylation assays. This same study revealed that, along with PKA, the cyclin-dependent kinase 1, casein kinase 2, glycogen synthase kinase 3, and p70 S6 kinase proteins are able to phosphorylate PXR in vitro [60].

The first direct line of evidence acquired that proved PXR is phosphorylated came from Lichti et al. using a radiolabeling experimental approach [46]. This study further revealed that modulation of threonine phosphorylation was achieved by cyclic AMP through PKA signaling pathways. It is noteworthy that PKA signaling interfaced very differently with PXR transactivation capacity in both rat and human liver cells, when compared with those from mice [47]. Increased PKA activity caused synergistic increases in *Cyp3a11* gene expression by PXR activation in cultured mouse hepatocytes. Conversely, increased PKA signaling produced robust decreases in PXR-mediated induction of *Cyp3A1* and *CYP3A4* gene expression in rat and human hepatocytes, respectively. Moreover, these authors discovered that PKA signaling produces an increased strength of association between PXR and the NCoR in cell-based assays, which may explain the strong repression in *CYP3A* gene expression in rats and humans when compared with mice. The biological significance of the polar opposite effects of PKA signaling in mice when compared with humans and rats is not well understood, although this remains a clear and rather unique observation. This is a potent reminder that rats are not humans, and humans are not mice with respect to their biology, per se.

A systematic analysis of predicted phosphorylation sites within the human PXR protein led researchers to hypothesize that multiple serine and threonine residues could potentially be impacted by kinase phosphorylation events on the PXR protein [46]. The authors used site-directed mutagenesis to study eighteen different predicted sites of serine (Ser) and threonine (Thr) phosphorylation. This study revealed that Ser-8, Thr-57, Ser-208, Ser-305, Ser-350, and Thr-408 were predicted to potentially have biochemical relevance in the regulation of PXR biology. It is now well established that phosphorylation of human PXR by PKC or PKA is associated with decreased transcription of its target genes in cell-based assays. Studies by Pondugula et al. have inferred a role for phosphorylation of Thr-57 by p70 S6 kinase to potentially regulate its DNA-binding activity [48]. Additional mutagenesis studies infer that PXR phosphorylation at Thr-248 and Thr-422 plays a role in modulating the ligand-mediated transactivation function of PXR [61].

More recent studies using direct methods to identify sites of phosphorylation have uncovered the specific regulatory roles of numerous protein kinases in the regulation of PXR biology. For instance, using a novel phospho-peptide antibody and Western blot analysis, Gotoh et al. showed that the Ser-350 residue of PXR was phosphorylated by VRK1, a member of the vaccinia-related kinase (VRK) family of serine/threonine protein kinases, in hepatocytes in conditions of low glucose. Recent studies have unveiled how phosphorylation of PXR at Ser-350 by VRK1 served as a molecular signal for increased hepatic gluconeogenesis and perhaps drug-mediated impairment of glucose tolerance in humans through activation of PXR [62,63,64]. The VRK1 protein has previously been shown to promote the stability and nuclear accumulation of a transcriptionally active p53 molecule and, in vitro, to phosphorylate Thr-18 of p53 and reduce p53 ubiquitination [65], providing a plausible paradigm for this important nuclear kinase in the regulation of PXR biology in response to glucose signaling.

An important recent research effort mechanistically linked PXR phosphorylation at amino acids Thr-135 and Ser-221 to co-regulatory protein-binding specificity. Using a novel adenovirus-mediated recombinant PXR expression, purification, and recovery system coupled with mass spectrometry, Cui et al. demonstrated that human PXR, directly recovered from cultured primary hepatocytes, was phosphorylated at Thr-135 and Ser-221 [66]. Phospho-mimetic mutation at Ser-221 (S221D) switched PXR corepressor protein partner preference from SMRT to NCoR. Moreover, the phospho-mimetic mutation at Ser-221 decreased the strength of interaction between PXR and transcription co-regulatory proteins RXRα, SRC1, SRC2, and PBP [66]. Studies by Elias et al. also directly identified Thr-135, among several other sites of phosphorylation, in cell-based assays using mass spectrometry [67]. Studies by Sugatani et al. revealed that phosphorylation of PXR at Thr-290 regulates the nuclear translocation of human PXR by Ca2+/calmodulin-dependent protein kinase II [51]. This same group has produced a more recent study which suggested that the degradation of human PXR is regulated through phosphorylation by PKC at Thr-408 to enter the CHIP/chaperone-dependent stability check and the cytoplasmic autophagy degradation pathway [68].

Taken together, these observations delineate that the response of PXR to modulation of its phosphorylation status can determine its biological function and suggest that further investigation is warranted to more fully understand the regulation of PXR by phosphorylation. Unlike the earliest studies of PXR phosphorylation, which relied heavily upon in silico phosphorylation prediction analysis of inferred consensus phosphorylation sites, more fruitful studies of this important NR family member should likely be accomplished using techniques that allow for the direct observation of PXR PTMs, such as mass spectrometry-based approaches, to substantially move the field forward.

### 2.2. SUMOylation

SUMOylation is a post-translational modification involved in the regulation of various pivotal biochemical processes that include nuclear-cytosolic transport, transcriptional regulation, apoptosis, protein stability, response to stress, and progression through the cell cycle [69]. Four small (approximately 11 kilodalton) SUMO proteins (originally termed sentrins) have been identified, which are termed SUMO1, SUMO2, SUMO3, and SUMO4 [69]. The structures of SUMO2 and SUMO3 are 97% homologous to each other. Hence, these two SUMO family members are often termed SUMO2/3 in the literature, and in fact have very similar biochemical function in many cell-based and in vivo biochemical assays. In contrast, SUMO1 is 45% homologous to SUMO2/3 and is therefore thought of as being functionally distinct from SUMO2/3. As with ubiquitination, SUMOylation pathways involve a cascade of enzymatic reactions which culminate in the attachment of a small ubiquitin-like modifier (SUMO) on key lysine (Lys) residues in its target substrate proteins. Target protein Lys residues can be modified by a single SUMO moiety, typically by SUMO1, or similar to ubiquitin, can form chains through an internal SUMOylation event on Lys-11 within SUMO2/3 on certain substrate proteins. Moreover, SUMO can be ubiquitinated to form mixed SUMO-ubiquitin chains. While the process of SUMOylation has many similarities with the ubiquitination process, the SUMO enzymatic cascade is distinct from the cascade responsible for ubiquitination.

The SUMOylation pathway starts with the heterodimeric E1 SUMO-activating enzyme (SAE1/SAE2). The activated SUMO molecule is then transferred to the E2 SUMO-conjugating enzyme (Ubc9). The E3 SUMO-ligase enzymes then engage with their respective target substrate proteins to complete the SUMOylation process [70,71]. In mammals, four SUMO E3-ligase genes in the protein inhibitor of activated STAT (PIAS) genes exist: PIAS1, PIAS2 (also known as PIASx), PIAS3, and PIAS4 (also known as PIASy) [72]. Very similar to the ubiquitination process, the SUMOylation cycle includes maturation of pre-SUMO protein through a proteolytic process that involves cleavage at its C-terminus by a family of sentrin proteases (SENPs), followed by activation through thioester bond formation, conjugation, ligation and de-modification by SENPs. There are six dedicated SUMO-specific members of the SENP family to include: SENP1, SENP2, SENP3, SENP5, SENP6, and SENP7 [73]. The family of SENPs are dual function proteases that both activate pre-SUMO, as well as detach SUMO moieties from SUMOylated target proteins in a SENP-specific manner.

Initial studies of PXR SUMOylation using in vitro methods revealed that human PXR could indeed serve as a substrate for SUMO1, SUMO2, or SUMO3 [74]. Cell-based analysis using cultured HeLa cells revealed that PXR was preferentially modified by the SUMO3 protein to form robust SUMO chains. To understand where the SUMOylation occurred on the PXR protein, experiments from two different research groups suggest that both K108 and K128/K129 serve as the principal SUMO attachment sites [56,58]. Additional cell-based experiments into the enzymatic cascade by Cui et al. revealed that PIAS1 and PIAS4 can both function as the most effective SUMO-E3 ligase enzymes for driving the SUMOylation of PXR, while SENP2 is the most effective de-SUMOylating SENP enzyme for all SUMO moieties on the PXR protein [56]. It is noteworthy that de-SUMOylation in the face of the SENP2 enzyme produced significant decreases in the levels of the PXR protein, suggesting that PXR SUMOylation is a stabilizing event.

In fact, rifampicin, the prototypical activator of PXR, has long been known to suppress immunological function in human hepatocytes [75]. It was hypothesized that the SUMOylation of PXR could play a role in this process [56]. Studies by these authors indicate that PXR is SUMOylated in response to treatment with ligand and tumor necrosis factor alpha (TNFα), which then functions to repress TNFα-inducible nuclear factor kappa-B (NF-кB) gene expression [74]. Using an adenoviral-mediated expression approach, co-transduction of human PXR and PIAS1 into *pxr* nullizygous cultures of mouse hepatocytes significantly repressed the induction of *TNFα* and *IL-6* gene expression levels in response to treatment with TNFα. These data could likely explain the mechanism through which PXR ligands modulate the inflammatory response in liver and intestinal cells, the primary sites of the highest levels of PXR expression in mammals.

### 2.3. Ubiquitination

**Figure 2:**
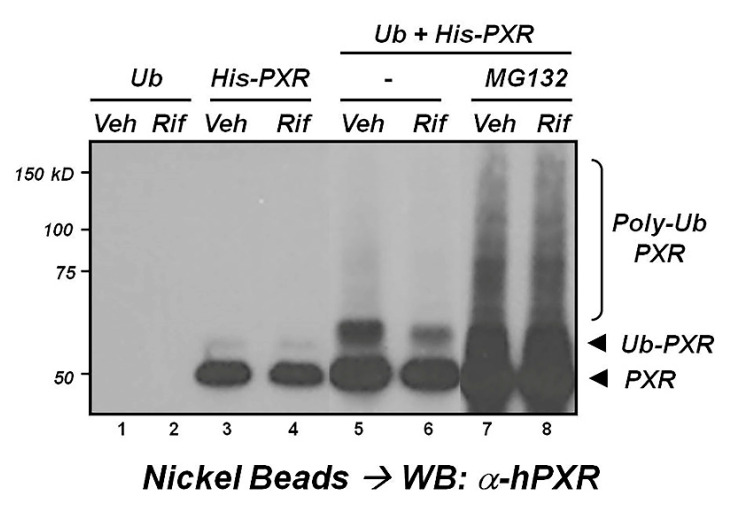
PXR is ubiquitinated in cells. The plasmid encoding 6x-Histidine-tagged PXR (His-PXR) protein was transfected alone or together with an expression vector encoding ubiquitin (Ub) into HeLa cells. Twenty-four (24) hours post-transfection, cells were treated for an additional 24 h (VEH = 0.1% DMSO, Rif = 10 μM, MG132 = 25 μM). Total cell extract was subjected to purification using nickel-linked agarose beads, followed by SDS-PAGE and subsequent Western blotting with anti-PXR antibodies (Santa Cruz-H11).

Ubiquitination is an important ATP-dependent PTM of numerous cellular proteins that include transcription factors, in particular NRs. In a signal-dependent manner, the ubiquitin protein is covalently attached to the side chain of a Lys residue in its target proteins. Following their ubiquitination, proteins are directed to the 26S proteasome for subsequent degradation [76]. Using a forced over-expression nickel bead-affinity purification approach, together with the pharmacologic inhibitor of the 26S proteasome, MG132, we show here that the ubiquitination of a 6X-Histidine-tagged form of PXR (His-PXR) can be directly demonstrated in transfected HeLa cells (Figure 2). Treatment of cells with cellular activators of PKA to include 8-bromo-cyclic AMP produces a profound increase in the detectable levels of ubiquitinated PXR [55]. Biochemical methods used to drive SUMOylation also promoted the ubiquitination of PXR, suggesting a pivotal interplay between the SUMOylation and ubiquitylation of PXR, possibly through mixed SUMO-Ub chain formation on Lys-128/Lys-129 [56]. In the same study, poly-ubiquitin chain formation of PXR on Lys-170 was directly observed using a mass spectrometry approach [56]. More recent efforts by this group indicate that Lys-109 and Lys-150 are also targets of ubiquitylation of the PXR protein [57].

Multiple E3 ubiquitin ligase enzymes have been identified that appear to drive the ubiquitination of PXR. In 2012, a yeast two-hybrid screen identified RANBP2-type and C3HC4-type zinc finger-containing 1 (RBCK1, also known as HOIL-1L) as a likely E3-ubiquitin ligase and PXR-binding protein [54]. The RBCK1 gene encodes a 58 kDa protein comprised of an *N*-terminal ubiquitin-like (UBL) domain, an Npl4-type zinc finger (NZF) domain, and a catalytic *C*-terminal RBR domain known as ring-B-box-coiled coil 1. It is noteworthy that RBCK1, in addition to binding and regulating PXR ubiquitination, also regulates NF-кB activity through its interface with the NF-кB essential modulator (NEMO) [77]. A 2014 study using a small interfering RNA (siRNA)-based screen of human kinases coupled with mass spectrometry analysis identified the ubiquitin protein ligase E3 component n-recognin 5 (UBR5) as another likely PXR-targeted E3 ubiquitin ligase [78]. Furthermore, this study revealed that the dual specificity tyrosine-phosphorylation-regulated kinase 2 (DYRK2) negatively regulates human PXR stability and activity. In their model, the phosphorylation of PXR by DYRK2 facilitates the ubiquitination of PXR by UBR5 through the formation of a multi-protein complex to regulate PXR homeostasis. These findings provide an additional mechanism whereby human PXR stability is regulated by kinase signaling-dependent ubiquitination of PXR.

**Figure 3:**
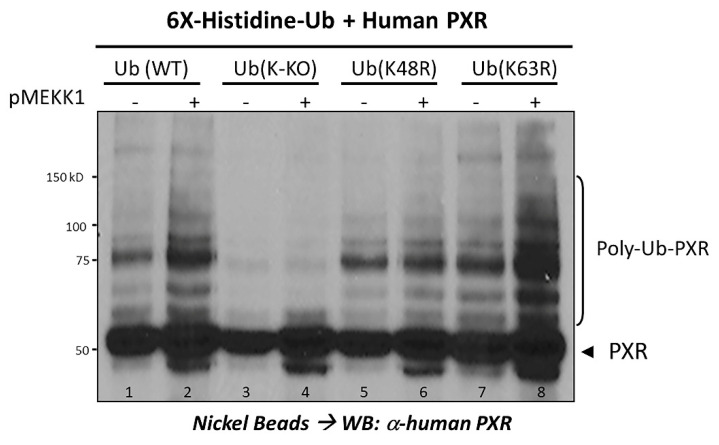
Poly-ubiquitination of the PXR protein is stimulated by the MEKK signaling pathway. The plasmid encoding the 6x-His-tagged ubiquitin protein and indicated mutant Ub expression vectors were co-transfected into HeLa cells with the expression vector encoding the PXR protein together with a constitutively active form of MEKK (a.a. 380–672), which activates JNK signaling cascade, as indicated. Total cell extract was subjected to purification using nickel-linked agarose beads, followed by SDS-PAGE and Western blotting using a monoclonal antibody against the human PXR protein (Santa Cruz, H-11).

The accepted canonical signal for proteasomal recognition is a poly-ubiquitin chain that is anchored to a Lys residue in the target substrate, and is assembled through isopeptide bonds involving Lys-48 (K48) of ubiquitin [79]. In previously unpublished data, we show here that the signal-dependent poly-ubiquitination of PXR via K48-linked chain formation on ubiquitin is stimulated by a constitutively active form of mitogen activated protein kinase kinase kinase (MEKK1) (a.a. 380–672) (Figure 3). These data are significant in that the MEKK signaling pathway links inflammation to NF-кB transcription, providing a plausible central molecular mechanism for the interface between drug metabolism (PXR) and the inflammatory signaling pathway (NF-кB) in the liver and intestines. In conclusion, PXR is able to be ubiquitinated at specific Lys residues to include Lys-109, Lys-150, and Lys-170. To summarize, the E3 ubiquitin ligases that have been identified to interact with PXR include UBR5, RBCK1, and TRIM-21. The ubiquitination of PXR is inextricably influenced by other PTMs such as phosphorylation, ADP-ribosylation, and SUMOylation. Taken together, these studies indicate that the ubiquitination of PXR is regulated at multiple levels, likely through its ability to be targeted for modification by several E3 ubiquitin ligase enzymes in response to key extracellular cell stimuli including xenobiotic-, inflammatory-, and nutrient-signaling pathways.

### 2.4. Acetylation

Protein acetylation is one of the major PTMs in eukaryotic organisms, in which the acetyl group from acetyl coenzyme A (Ac-CoA) is attached to a specific site on a newly formed polypeptide chain. In humans, it has been speculated that 80–90% of all proteins become co-translationally acetylated at their *N*-termini [80]. Another distinct type of protein acetylation targets the epsilon-amino group of Lys residues in specific target proteins [81]. Initially characterized in histone proteins, it was speculated that acetylated-Lys residues were heavily involved in regulating gene activation; thus, the family of enzymes catalyzing Lys acetylation were termed histone acetyltransferases. Nevertheless, Lys-acetylation is not exclusively limited to histone proteins, and the enzymes that catalyze protein acetylation have been renamed lysine-acetyltransferases (KATs) [82]. A large family of KATs has been identified that function to attach acetyl groups to Lys residues on specific subsets of target proteins [83]. Likewise, histone-deacetylase enzymes are now referred to as lysine-deacetylases (KDACs) to more accurately describe their function of removing acetylation on numerous non-histone proteins, to include many transcription factors. The KDAC superfamily of proteins is grouped into four separate classes based on their function and DNA sequence similarity. Class I, II, and IV are considered “classical” HDACs whose activity can be pharmacologically inhibited in cells by trichostatin A (TSA). Class I, II, and IV also possess a zinc-dependent active catalytic site. The class III KDAC enzymes comprise a distinct sub-family of NAD^+^-dependent proteins known as sirtuins. Sirtuins are unique in that they are totally unaffected by treatment with TSA [84].

A recent study suggests that alterations in the acetylation status of the PXR protein has a profound effect on its transactivation capacity. Biswas et al. revealed that PXR is directly acetylated in a transfected cell line [85]. Ligand-mediated activation of PXR reduces the levels of acetylation, and the deacetylation of PXR is mediated, at least partially, by a class III NAD^+^-dependent KDAC called SIRT1. Another study from this group identified p300, well-known for its ability to deacetylate numerous NRs, as a likely KDAC enzyme that removes acetyl groups from Lys residues within PXR [86]. Moreover, ligand-induced activation of a PXR expression vector containing an acetyl-mimetic mutation at the Lys-109 position (K109Q) reduced levels of PXR-transactivation capacity in response to treatment with SR12813, a well-known human PXR activator. The p300 KAT enzyme regulates transcription by directly binding to NRs through a well-defined NR-interaction domain (NRID) [87].

Additional insights into the role of PXR acetylation revealed that pharmacological inhibition of PXR deacetylation with TSA reduced the induction of PXR-target genes [57]. In addition, this study revealed an acetylation-dependent multi-protein complex comprised of PXR, SMRT, and HDAC3, a well-defined NR co-repressor complex. Of note, the study by Cui et al. also revealed a reciprocal effect of PXR SUMOylation and acetylation.

In another study, Bakshi et al. revealed that PXR is also acetylated on Lys-170 by KAT5, a well- known KAT protein (also called TIP60), that is involved in regulating chromatin remodeling, transcription, and other nuclear processes by acetylating histone and non-histone proteins. In this study, it was revealed that KAT5 utilizes its NR-box to interact with the LBD region of PXR and acetylates PXR at Lys-170 to induce its intra-nuclear reorganization [88]. Interestingly, these authors observed that PXR interaction with TIP60 augments the catalytic activity of TIP60 towards the acetylation of histone proteins. It is therefore abundantly clear that the acetylation of PXR plays a key role in defining PXR transactivation capacity and altering its biological function.

PXR has been shown to have a role in major metabolic processes within the body, including lipid metabolism, drug metabolism, and gluconeogenesis [66,89,90]. Furthermore, nutrient excess conditions have been shown to promote acetylation levels of metabolic regulators, including NR proteins specifically, which may suggest the physiological relevance of PXR acetylation in patients with obesity and other metabolic disease processes. Further research efforts should be focused on gaining insight into the role of interplay between PXR acetylation and chronic metabolic disease pathologies and linking that to possible therapeutic approaches.

### 2.5. Poly(ADP-Ribosyl)ation

Poly(ADP-ribosyl)ation, also known as PARylation, is a PTM in which polymers of ADP-ribose (poly(adenosine diphosphate-ribose)) are covalently attached to proteins by PAR polymerase (PARP) enzymes in the ADP-ribosyltransferase (ART) superfamily [89]. ADP-ribosylation is characterized by the addition of ADP-ribose from NAD^+^. Poly(ADP-ribosyl)ation has classically been studied in immediate DNA-damage-dependent PTMs of histones and other nuclear proteins that contribute to the survival of injured proliferating cells. In a signal-dependent manner, the polymerase enzymes covalently attach poly(ADP-ribose) polymers to themselves and appropriate acceptor proteins to include histones and other DNA-binding proteins. ADP-ribosylation is best characterized to regulate chromatin organization, DNA repair, transcription, replication, and other biochemical processes. The role of PTM via ADP-ribosylation has been established in DNA damage signals, DNA double-stranded breaks in response to cellular toxicity, the regulation of cell cycle checkpoints, and apoptosis pathways. Notably, the addition of ADP-ribose is not a discrete event; rather, it is part of a symbiotic and combinatorial relationship between post-translational modifiers and its effects have yet to be fully elucidated. The PARP enzymes have been more extensively studied within the ART superfamily and are best studied in PARP-1. The PARP-1 protein is implicated in the transcriptional activity of NF-kB and its subsequent downstream inflammatory effectors [90]. The relationship between PARP-1 and NF-kB was first studied in the context of septic shock. PARP-1 deficient mice were exposed to the bacterial component lipopolysaccharide (LPS), a known inducer of NF-kB transcriptional activity. Due to LPS, the PARP-1 null mice were found to be resistant to septic inflammatory cascade associated with NF-kB, suggesting that PARP-1 plays a role in inducing NF-kB [90]. In contrast to NF-kB activity modulation, ADP-ribosylation of PXR is not currently as well characterized in the literature; however, a key interface between drug metabolism and inflammation is well-known [91,92,93,94,95].

Poly(ADP-ribosyl)ation of PXR has been solely explored by Wang et al. [59]. The regulation of acetaminophen (APAP) metabolism and toxicity by PXR activation is well studied, and therefore provides good scaffolding to explore the effects of ADP-ribosylation [96]. *N*-acetyl-p-benzoquinone imine (NAPQI), also known as NAPBQI, is a toxic byproduct produced during the CYP3A-mediated xenobiotic metabolism of the analgesic paracetamol (acetaminophen). Using a mouse model, Wang et al. revealed that PARP-1 was responsible for ADP-ribosylation of PXR in the context of PXR-activators to increase APAP metabolism. PARP-1 was activated in the presence of APAP and subsequently ADP-ribosylated PXR. ADP-ribosylated PXR up-regulates *Cyp3a11* gene expression, thereby increasing APAP hepatotoxicity via increased metabolism to NAPQI. The interaction between PXR and PARP-1 was found to be within the BRCA-C terminus at the auto-modification domain of PARP-1 and the ligand-binding domain (LBD) of PXR. The PXR-LBD is where ADP-ribosylation occurred [3]. The finding that activation of PXR causes up-regulation of Cyp3a11 mRNA and a subsequent increase in sensitivity to hepatotoxicity is congruent with prior studies [96,97]. However, PXR (−/−) mice have been reported to have a 3-to-4-fold greater expression of *Cyp3a11*, yet decreased sensitivity for hepatotoxicity, suggesting that the role of PXR on cytochrome P450 induction is complex and accessory protein cofactor crosstalk is present [16,97].

**Figure 4:**
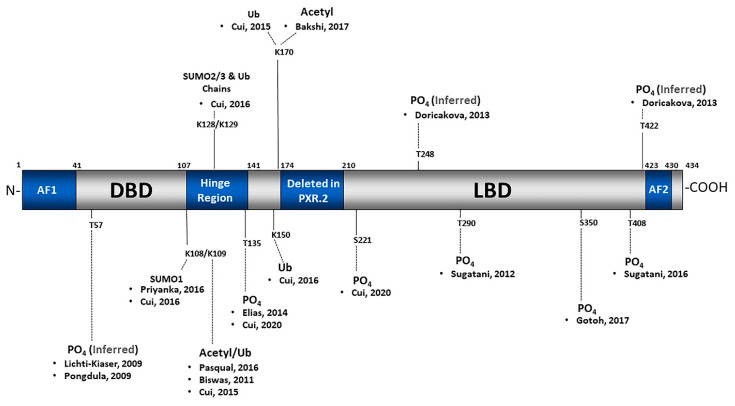
This scheme provides the specific sites and types of PXR PTMs and their associated references.

The role of ADP-ribosylation in the regulatory networks of PXR and NF-kB activation is stunning and clinically important. The strong inverse relationship between drug metabolism pathways and the inflammatory response suggests an interesting and important interconnected regulatory mechanism involving ADP-ribosylation that needs to be better characterized and studied [98]. This interconnection likely involves coordination with different PTMs as reported in other cellular processes [99]. A scheme representing the PXR protein and its associated PTMs are annotated with the respective studies in Figure 4.

## 3. Conclusions

Cells employ a complex network of molecular pathways to cope with endogenous and exogenous biochemical and xenobiotic stress. Signal-activated ubiquitination and SUMOylation pathways lead to extensive chain-like modifications on the PXR protein, a feature that is shared with poly(ADP-ribosyl)ation (i.e., PARylation). This multi-layered response ensures that gene–environment stressors are efficiently detected and appropriately responded to, in order to safeguard our bodies’ needed biochemical, nutritional, inflammatory, and pharmacological homeostasis. The molecular choreography that is activated following xenobiotic, inflammatory, and nutritional stress relies heavily on PTMs. Protein modifications with ubiquitin and the small ubiquitin-like modifier SUMO have recently emerged as important regulatory means to inversely coordinate inflammation and drug metabolism pathways. In addition, chains of ubiquitin, SUMO, and PAR all contribute to the assembly of multi-protein complexes found at genomic PXR-binding sites of drug-inducible gene expression in the liver and intestines through alterations in PXR PTM status in order to regulate specific temporal and spatial aspects of PXR-mediated gene activation. Here, we review recent advancements in our understanding of how phosphorylation, ubiquitylation, SUMOylation, and PARylation coordinate the xenobiotic response and highlight emerging examples of an intricate interplay between these chain-like modifications during the cellular response to potentially toxic environmental stress.

### 3.1. PXR and SUMOylation

It has been widely held that SUMOylation of different transcription factors leads to transcriptional repression [100]. The current knowledge paradigm of transcriptional suppression holds that SUMOylation results in the recruitment of histone deacetylase enzymatic activity to target promoters. In particular, HDACs are thought to be recruited to these promoters following SUMOylation through novel protein–protein interactions. This recruitment leads to increased histone deacetylation, and hence, transcriptional repression at NR-specific target genes. This paradigm suggests an important integration point for two protein-modifying pathways in the cell, the SUMO and deacetylation pathways, that combine to promote transcriptional repression. Cui et al. observed that the introduction of human PXR along with PIAS1, an E3 SUMO-protein ligase, into PXR-KO mouse hepatocytes significantly increased the ligand-inducible transactivation of *Cyp3a11* gene expression, when compared to transduction with human PXR alone [56]. Priyanka et al. observed similar results when they found that transfection of the HepG2 cell line with SUMO1 significantly enhanced the transcriptional activity of PXR towards rifampicin-mediated induction of CYP3A4 in a dose-dependent manner. In contrast, using modified PXR-SUMO fusion protein expression vectors that directly fuse SUMO1 or SUMO3 to PXR indicate that this form of linear SUMO-PXR fusion protein produces a strong repression of its transactivation capacity in reporter gene analysis [57]. The covalent attachment of SUMO to the *N*- or *C*-terminus of PXR examines the direct effect of SUMO-modified PXR, when compared to the natural isopeptide linkage that occurs when PXR is SUMOylated through the normal endogenous enzymatic cascade stimulated SUMOylation by directly increasing levels of PIAS1, PIASy, SUMO1, and SUMO3, respectively [56,58,101]. In addition to increased SUMOylated PXR, the SUMOylation of many co-regulatory proteins is also likely to be increased in these experiments. Future efforts should focus on the differential effects of PXR SUMOylation upon direct PXR-target gene expression versus trans-repression of inflammatory response genes to elucidate molecular mechanisms of these two important intertwined pathways.

### 3.2. A SUMO-Acetyl Switch

A direct correlation was noted between PIASy-driven SUMO(1)ylation of PXR and levels of ubiquitinated PXR, suggesting that SUMO-modification of PXR increases ubiquitylation, and likely mixed SUMO-ubiquitin chain formation. Importantly, both the acetylation and SUMOylation status of the PXR protein affect its ability to associate with the KDAC3/HDAC3 in a complex with the SMRT corepressor protein. Co-immunoprecipitation of PXR together with KDAC3 is abolished following PXR SUMO(3)ylation by PIAS1. However, inhibition of PXR deacetylation with TSA restores the interaction with HDAC3 in the face of PIAS1 and SUMO3 [57]. These data suggest that the PXR-co-repressor multi-protein complex is acetylated and poised for gene activation, forming a “gene-off” posture. The acetylation of PXR marks this NR protein as “competent” to become SUMOylated to thereby drive SUMO-PXR-dependent transrepression processes through protein–protein interactions with other transcriptional machinery, or to form ubiquitin-PXR to drive a round of canonical PXR-target gene expression. Overall, the data support a model in which a SUMO-acetyl switch occurs such that acetylation likely marks PXR as competent to fire a round of transcription, or to acquire SUMO-modification to drive transrepression of NF-кB target gene expression, likely through SUMO-dependent processes.

### 3.3. PXR, Phosphorylation, and Drug Metabolism

A recent study indicates that PXR activation inhibits platelet function, and that effect is associated with the inhibition of the Src-family of kinase enzymes, strikingly, in the absence of a nucleus [102]. This study therefore has identified rapid and non-genomic regulatory effects of PXR activation on basic platelet function and subsequent thrombus formation. We suggest that PXR activation with agonists in platelets could provide additional cardio-protective benefits for heart attack patients by preventing inappropriate thrombus formation. It is theoretically possible that PXR activation can be achieved outside of the ligand-dependent manner described here, though it has yet to be demonstrated for this pivotal NR family member whether all PXR activation is ligand-dependent, or is a response to phosphorylation, per se.

Both the PKA- and PKC-signaling pathways have been definitively shown to reduce CYP3A gene expression in hepatocytes when active. However, it is still largely unknown if they do so through phosphorylation of PXR and their total target sites of phosphorylation on the PXR protein. It is abundantly clear that there are numerous sites of phosphorylation on PXR, and the regulation of PXR biology is thought to involve its phospho-modification at multiple sites through numerous signaling pathways.

It is of note that important pro-inflammatory cytokines including interleukin-1, interleukin-6, and TNFα can activate PKC signaling, suggesting a direct and suppressive effect of PXR and its target genes during inflammatory conditions. It is also important to note that hepatocyte growth factor decreases PXR expression, meaning during periods of liver regeneration CYP expression will be decreased. Increased levels of CDK2 also produce a decrease in CYP3A4 and CYP2B6 gene expression in human hepatocytes. Taken together, CYP expression and drug metabolism are reduced during inflammation and the primary mechanism through which PXR activity is repressed is through its phosphorylation [103].

### 3.4. PXR and Glucose Metabolism

There are many physiological associations that PXR phosphorylation has in conjunction with chronic metabolic diseases. PXR activation will enhance glucose levels in the body, both directly and indirectly. It has been shown that statin-activated PXR caused depressing effects on SGK2, a gluconeogenesis gene repressor [102]. Activated PXR is effectively repressing a repressor of gluconeogenic genes. In addition, activated PXR also interacts with the FOXO1 gene, decreasing the expression of the insulin receptor substrate gene (IRS), decreasing insulin receptor signaling [104]. It is important to note that PXR is working in tandem with another nuclear receptor, the constitutive androgen receptor (CAR), to decrease IRS expression. Therefore, activated PXR can have an impact on serum glucose levels and is thus implied in glucose-associated metabolic diseases, such as type 2 diabetes mellitus. Further research and data are needed in this area of glucose-associated diseases and PXR. However, through understanding the inhibitory effects of phosphorylation on PXR, there could be avenues of exploration in the prevention and treatment of these diseases through this mechanism.

### 3.5. PXR and Lipid Metabolism

Molecular analysis of PXR’s effects on lipid metabolism shows that Rif-PXR activates the SLC13A5 gene, which will translate a transport protein that allows citrate, a fatty acid precursor, into the mitochondria of human hepatocytes [105]. Excess activation could possibly lead to the accumulation of too much citrate and consequently accumulated fatty acids. Therefore, it is hypothesized that PXR activation plays a role in lipid storage diseases, such as hepatic steatosis. It is unclear the impact PXR activation plays in hepatic steatosis, but it is theorized that the phosphorylation of PXR could negatively contribute to the accumulation of lipid in liver tissue.

### 3.6. PXR and Inflammation

The interplay between PXR and NF-кB has been well described in the literature [74,91,98,106,107,108,109,110,111,112,113]. The balance between these two proteins allows for negative crosstalk between drug metabolism and inflammation. During inflammatory conditions, PXR-target gene expression is remarkably decreased. Conversely, PXR activation produces a repression of inflammatory genes. This phenomenon can be exploited to treat inflammatory conditions, most historically inflammatory bowel disease [95,96,114,115]. A further thrust of research is needed in this field in order to fully elucidate the molecular mechanisms of this phenomenon; however, SUMOylation of PXR and its heterodimeric partner RXRα’s ability to interact with NF-кB undoubtedly play a role.

### 3.7. PXR and Cancer

Clinically, much is still left to be uncovered concerning the effects PTMs have on PXR influenced drug–drug interactions. Because PXR is a direct substrate for phosphorylation by CDK2 the use of potential therapeutic cyclin-dependent kinase inhibitors is expected to cause a significant decrease in CYP3A4 and P-glycoprotein [62,63,102]. This fact suggests that the adverse effects associated with cancer pharmacotherapy using cyclin-dependent kinase inhibitors are related to PXR biology. Given the vast number of kinases associated with the phosphorylation of PXR, additional research is required to understand the precise molecular mechanisms behind each. Essential kinases, such as CDK2, are well understood to this point as elucidated in this theme issue. However, a greater understanding of many others is required to help formulate better dosing regimens for many promising drugs for treating metabolic issues, inflammatory issues, and cancers.

In the context of certain types of cancer, PXR activation regulates cell proliferation, metastasis, apoptosis, anti-apoptosis, inflammation, and oxidative stress differentially depending on cancer cell-types that include prostate, breast, ovarian, endometrial, and colon cancer [116]. PXR activation also induces differentiation of osteoblasts and apoptosis of osteoclasts and certain leukemia cells [114,115,117,118,119]. These studies indicate that control of cell proliferation and cell differentiation following PXR activation is very likely tissue- and cell-type specific. A similar theme plays out in cancer cells in which PXR differentially regulates cell growth through multiple mechanisms in a variety of cancers that include liver, prostate, breast, ovarian, endometrial, cervical, and colon. Additionally, PXR is involved in regulating the metastatic phenotype of various cancer cells [120,121,122]. PXR also plays a key role in chemotherapeutic resistance outcomes in specific cancers that include breast, prostate, endometrial, ovarian, and colon cancers [116,122,123,124,125,126,127,128]. By regulating the expression and activity of enzymes and proteins involved in drug metabolism, drug transport, proliferation, apoptosis, anti-apoptosis, inflammation, and oxidative stress, PXR activation in response to certain chemotherapeutics promotes the acquisition of resistance to anti-cancer agents in a specific subset of tumors.

Taken together, these data indicate that PXR plays a differential role in regulating the behavior of cancer cells, and the tumor type plays a very important role. It is clear that additional research efforts are needed, and moving forward, investigators should likely focus on the differential role of PXR biology, particularly in the context of disease states and various pathophysiological conditions. It is worth stating here that “proper” or “normal” activation of PXR in non-tumorigenic cells is likely to be very different from a mechanistic standpoint when compared with “over/sub-activation” of PXR in diseased states. Future studies should seek to unravel these mechanistic differences moving forward.

### 3.8. PXR-Target Gene Activation and Poly(ADP-Ribosyl)ation (PARylation)

The formation of poly(ADP-ribose) chains on the PXR protein likely functions to attract a diverse set of PXR-accessory proteins. PARylation of PXR via PARP1 in response to xenobiotic stress ultimately leads to chromatin remodeling. Additional studies have demonstrated the role of poly(ADP-ribosyl)ation in the ubiquitin pathway. One such study published in 2016 demonstrated that PARylation may serve as a recognition signal for selected ubiquitin ligases [99]. Further research is needed to unravel the precise role of PARylation in these processes.

### 3.9. Summary

The interplay of acetylation, ubiquitylation, SUMOylation, and PARylation establishes a likely temporal and spatial mechanism to drive the xenobiotic response. These interconnected signaling pathways frequently use positive feedback loops to amplify the signal and further increase its specificity. It is currently unclear whether, or the extent to which, other PTMs impact PXR biology when compared with other NRs to date. For example, *O*-linked glycosylation and neddylation are known to affect the biology of other NR family members; however, more research is needed to ascertain if PXR is the molecular target of these PTMs at present. Indeed, much remains to be discovered regarding the precise molecular mechanisms of how phosphorylation, SUMOylation, ubiquitylation, and acetylation signaling are intricately intertwined to help remove potentially toxic compounds from the human body. What is clear is that phosphorylation-dependent mixed SUMO-ubiquitin chain formation on PXR and its overall acetylation status all occur on Lys amino acid residues and have a particular effect upon the metabolism and transport of xenobiotic compounds in the human body.
